# Is This My Own Body? Changing the Perceptual and Affective Body Image Experience among College Students Using a New Virtual Reality Embodiment-Based Technique

**DOI:** 10.3390/jcm8070925

**Published:** 2019-06-27

**Authors:** Bruno Porras Garcia, Marta Ferrer Garcia, Agata Olszewska, Lena Yilmaz, Cristina González Ibañez, Mireia Gracia Blanes, Gamze Gültekin, Eduardo Serrano Troncoso, José Gutiérrez Maldonado

**Affiliations:** 1 Department of Clinical Psychology and Psychobiology, University of Barcelona, Passeig de la Vall d’Hebron, 171, 08035 Barcelona, Spain; 2 Child and Adolescent Psychiatry and Psychology Department, Hospital Sant Joan de Déu of Barcelona; Passeig de Sant Joan de Déu, 2, 08950 Esplugues de Llobregat, Barcelona, Spain; 3 Children and Adolescent Mental Health Research Group, Institut de Recerca Sant Joan de Déu, Passeig de Sant Joan de Déu, 2, 08950 Esplugues de Llobregat, Barcelona, Spain

**Keywords:** virtual reality, body image disturbances, body anxiety, fear of gaining weight, full body illusion

## Abstract

Body image disturbances (BIDs) have been widely studied using virtual reality (VR) devices that induce a full body illusion (FBI) and allow manipulation of the individual’s perceptual and affective experiences of the body. This study aimed to assess whether the induction of the FBI over a virtual body would produce changes in body-related anxiety and BIDs using a new whole-body visuo-tactile stimulation procedure. Fifty non-clinical participants were randomly assigned to synchronous or asynchronous visuo-tactile groups. During the pre-assessment, all participants filled in BIDs and body-anxiety questionnaires. Then, they were embodied into two virtual bodies (VBs): firstly, with their real measurements, and secondly, with a larger-size body. Body image disturbances, body anxiety, fear of gaining weight, and FBI levels were assessed after exposure to each avatar. All participants in both conditions showed higher levels of BIDs and body anxiety after owning the larger-size VB than after owning the real-size VB (*p* < 0.05). The synchronous visuo-tactile group had higher scores, although the differences did not reach statistical significance. This study provides evidence of the usefulness of this new embodiment-based technique to induce changes in BIDs or body anxiety in a non-clinical sample, being suitable for use in future body image interventions.

## 1. Introduction

Body image has been described as a multi-dimensional construct reflecting the mental representation a person has of their physical appearance [1], including perceptual, cognitive, attitudinal, and affective components [2,3]. Likewise, body image disturbances (BIDs) involve dysfunctional cognitions, attitudes, and emotions related to the way in which an individual experiences their own body shape or weight. Body image distortion (the perceptual component) and body image dissatisfaction (the affective component) are the most commonly studied and assessed BIDs [4,5]. Body image distortion refers to the difficulty of precisely estimating one’s own body size, whereas body image dissatisfaction refers to the degree a person likes or dislikes their own body [6]. Body image disturbances have also been related to avoidance behaviors and negative checking strategies [7]. 

Other studies have suggested that body image may be a state rather than a trait and may, therefore, be modifiable, depending on the situation or emotional variables [6,8]. One emotional variable directly related to BID is fear of gaining weight and the resulting state body anxiety. Similarly, an excessive concern with having a thinner body is related to high anxiety levels, and that, consequently, affects BID [9]. 

Body image disturbances have a high prevalence among eating disorder patients, as well as in non-clinical populations. Regarding body dissatisfaction, previous studies found that 44% of women and 17% of men in European adult samples would like to lose weight [10]. In Spanish college students, 84.2% of young women were not satisfied with their physical appearance and that 70% of women and 52.8% of men desired to reduce their weight [11]. In relation to perceptual body image distortion, previous studies found that both eating disorder (ED) patients and healthy individuals overestimated their BMI by more than two points [12]. 

Virtual reality (VR) has been successfully used in the assessment and treatment of BID [13]. This technology allows researchers to create real-size 3D simulations of participants’ bodies with their specific physical characteristics and place them in VR immersive environments that reproduce real-life situations related to their body image concerns [13,14]. Recent studies have progressed to assessing the effect of inducing the ownership illusion of a virtual body on the experience of one’s own body (e.g., [15,16,17,18]). To do so, the avatar must elicit feelings of ownership and the participants must recognize themselves in the virtual avatar [17]. 

To induce these illusory feelings of ownership, it is necessary to synchronize the stimulation of the real body and the false body using different sensory modalities (e.g., visual, tactile, motor, and vestibular) [18]. Visuo-tactile synchronization is one of the most widely studied and best-known methods of eliciting this full body illusion (FBI) and is based on the paradigm of the rubber hand illusion (RHI) [19]. In this paradigm, the participant was first seated in front of a rubber hand which was positioned in the place of their real hidden hand. Next, the participant’s non-visible hand was touched as they saw the fake rubber hand being touched synchronously, thus eliciting an illusion of ownership over the fake rubber hand. According to Botvinick and Cohen [19], asynchronous tactile and visual stimulation would significantly reduce this illusion. 

Immersive VR devices have been widely used to investigate several important aspects related to FBI, such as the type of multisensory stimulation applied to produce it (e.g., visuo-tactile versus visuomotor stimulation) [20], or the sort of visual perspective from which the virtual body is observed (first versus third person perspective) [16]. Regarding the type of multisensory stimulation, there is no definite agreement in the literature at present about the best method to elicit the illusion. Several authors suggested that this illusion can be elicited using a synchronous visuo-tactile stimulation, in which the participant observes the virtual body or some specific virtual body parts being touched while also feeling touches on their own real body [15,18,21,22,23]. Other studies have also elicited the FBI by performing synchronous visuomotor stimulation [20,24,25]. Therefore, the results suggest that both visuomotor and visuo-tactile synchronous stimulation can induce the FBI [20], and that the illusion can be strengthened by combining vision, touch, motor control, and proprioception inputs in the same interventions [26]. 

Regarding the role of visual perspective, participants can either look at themselves in a first-person perspective (1PP) or in a mirror perspective, by observing their virtual bodies in a mirror. While some studies suggest that 1PP is necessary for eliciting FBI [21,25,27], others have also induced it by using a mirror view [16] or third person perspective [28]. Therefore, not only first-person visual perspectives can play an important role in creating the FBI [16]. 

A considerable number of studies have investigated the role of the FBI in attempts to modify body image disturbances (for extensive recent reviews, see Gutiérrez–Maldonado et al., 13,14), by changing the perceptual experiences of the size and shape of certain body parts [16]. For instance, the FBI was elicited in participants within a virtual avatar with enlarged arms [29]. Similarly, in another study participants felt an enlarged virtual abdomen as their own [22]. In a non-clinical sample, the FBI was elicited in a virtual avatar whose body mass index had been previously modified to have an enlarged or a thinner body [17]. 

Preston and Ehrsson [15] elicited the FBI in individuals who were exposed to a virtual mannequin body that was manipulated to be slimmer or wider than the individual’s real body. In addition, they achieved significant reductions in individuals’ perceived body size and increases in body satisfaction levels when participants owned a slim mannequin body. Similarly, Serino et al. [18,30] showed how non-clinical participants [18] and a single case patient with anorexia nervosa (AN) [30], reported significant reductions in the ratio between estimated and actual measurements of body parts after they were embodied into a VR avatar with a skinny abdomen. In a study with a clinical sample, Keizer et al. [23] found that owning a skinny virtual body significantly reduced the perceived body size in AN patients as well as in healthy participants. Additionally, this change in perceived body size was preserved two hours later, especially among the healthy participants. Finally, two previous studies with healthy samples found that owning a larger-size virtual body can produce changes in body representations [22] and can also elicit higher body anxiety levels [31]. Therefore, the use of FBI in VR environments may be a suitable method for manipulating the perceptual and emotional experiences of the body image.

In order to extend our knowledge of the usefulness of VR techniques to modify body image disturbances and body-related anxiety, this study presents a novel VR embodiment-based technique. Among the novelties of the current study are: the use of a visuo-tactile stimulation procedure over the whole body, in both 1PP and mirror view; the reproduction of a real-size virtual body, based on each participant’s frontal silhouette, resulting in accurate representations of their actual body; and a realistic simulation of their weight gain through different body parts (e.g., stomach, hips, waist, etc.) based on their real silhouette. 

The main aim of the study was to assess whether the induction of the FBI of a virtual body produced changes in body-related anxiety and body image disturbances of non-clinical participants. Participants owned two different virtual bodies (VBs): one with the same body size as their own and another with a larger body size. In addition, participants were randomly assigned to the synchronous or asynchronous visuo-tactile condition. 

Based on previous studies (e.g., [22,31]), we expected: first, that owning a larger-size VB would elicit higher body anxiety and body image disturbances than owning the real-size VB in both synch/asynch groups (and that the synchronous group would have higher scores); second, that the visuo-tactile synchronous group would show higher FBI levels in the real-size and larger-size VBs than the asynchronous group. 

## 2. Materials and Methods

### 2.1. Participants

This study was approved by the ethical committee of the University of Barcelona. The sample was composed of 50 undergraduate students at the University of Barcelona, recruited through campus flyers and advertisements in social network groups. Forty women and 10 men (M_age_ = 21.8, SD = 2.55, M_BMI_ = 22.5, SD = 2.51) participated in the study. Exclusion criteria were self-reported diagnosis of a current ED, a BMI under 17 (moderate thinness) or over 30 (obesity) according to the World Health Organization [32] or a current self-reported severe mental disorder diagnosis (e.g., schizophrenia or bipolar disorder). Each participant was given an identification code in order to guarantee the confidentiality of the data. 

### 2.2. Measures

#### 2.2.1. Assessment of Body Image Disturbances and Body Anxiety

Figural Drawing Scale for Body Image Assessment (BIAS-BD) [12]: This questionnaire allows the use of physical anthropometric dimensions of adult women and men by providing a series of human silhouettes. This questionnaire has two versions (A and B) in which silhouettes are randomized differently to avoid order effect bias. Participants selected, among a set of human silhouettes, the one that was perceived as their body size (perceived silhouette) and the one that was desired to have (desired body size). Then, according to their BMI, the real silhouette was also selected. Body dissatisfaction (BIAS-O) was assessed by calculating the discrepancy between the perceived and desired body sizes. Body distortion (BIAS-X) was assessed by calculating the discrepancy between the perceived and real body sizes. This questionnaire presents good psychometric properties, with a good reliability test-retest (*r* = 0.86) and a good concurrent validity (*r* = 0.76) [12].

Physical Appearance State and Trait Anxiety Scale (PASTAS) [33]: This body anxiety questionnaire comprises two separate self-report scales which measure weight-related and non-weight-related anxiety. In this study, we used the weight scale (W). This questionnaire presents good internal reliability, with a Cronbach’s alpha that ranges from 0.82 to 0.92, good test–retest (*r* = 0.87) and good convergent validity indices for the W scale (*r* = 0.74 EDI-BD, *r* = 0.62 EDI-DT) [34]. 

Visual Analog Scale (VAS)-FGW: This scale ranging from 0 (zero) to 100 (complete) assesses the fear of weight gain that the individual is feeling at a specific moment. The following question was posed to the participants: “On a scale from 0 to 100, rate how afraid you are of gaining weight right now”.

#### 2.2.2. Assessment of the Full-Body Illusion (FBI)

VAS-FBI: This visual analog scale ranging from 0 (zero) to 100 (complete) assesses the level of FBI of the virtual body that the individual is feeling at a specific moment. The following question was posed to the participants: “On a scale from 0 to 100, indicate to what extent you felt that the virtual body was your own body”. In case a participant asked about the meaning of the previous question, it was clarified that the expression “feeling the virtual body as your own body” referred to whether they had the sense of being the owner of the virtual body. 

### 2.3. Hardware and Software Features

Participants were exposed to an immersive virtual scenario using a head-mounted display (HMD) HTC-VIVE connected to a computer with enough graphic and processor power to move VR environments. Two programs were used to develop the virtual simulations: Blender 2.78 to create the virtual avatars (a man and a woman), and Unity 3D 5.5 to integrate all the elements within a virtual environment, which consisted of an unfurnished room with a large mirror placed in front of the participant’s avatar, 1.5 meters away. It was not visible at the beginning of the experiment (first-person perspective) and was activated only during the mirror view condition. In the back part of the room there was a small door, slightly open, which was placed there to avoid any feeling of being trapped inside.

Both male and female avatars wore a standard black t-shirt with black jeans and black trainers, as well as a swimming cap to reduce the idiosyncratic influence of hairstyle.

### 2.4. Procedure

Pre-assessment: Once they signed the informed consent form, all participants completed a test battery which included the BIAS-BD and PASTAS trait and state questionnaires. All participants were weighed and measured after completing the two questionnaires in order to avoid the possibility that anxiety might affect the questionnaire responses. The BMI was calculated using the classical formula (BMI = weight (kg)/height (m)^2^).

Creating the real-size virtual avatar (whole body photograph): The first step in creating the virtual avatar with the real measurements of the participants was to take a frontal view photograph of the whole body of the participant. A high-definition (HD) Logitech camera was used. All participants had to remain still in a marked position, two meters away from the camera, with their arms slightly raised and their legs slightly separated. Once the participant’s photo was taken and processed in our program, the experimenters manually overlapped the photo and the virtual body by adapting the height and different body measures of the virtual avatar (e.g., arms, legs, hip, waist, chest, shoulder, etc.) to the silhouette of the participant. The resulting virtual avatar, which represented the participant’s frontal body measurements, will be referred to from now on as the “real-size virtual body”. Finally, all participants were located within the avatar’s body during the exposure to an immersive VR environment. 

Visuo-tactile stimulation procedure: To enhance the illusion of owning the virtual body, an adaptation of the visuo-tactile stimulation procedure used by Keizer et al. [23] was conducted. While the Keizer et al. [23] procedure consisted of applying a tactile stimulation only to the abdomen for 90 seconds, in our study we also induced tactile stimulation over other specific body parts (the upper and lower limbs). Therefore, a series of continuous touches were applied to specific body parts, with a total duration of a minute and a half (15” each for left and right arm, 30” to the abdomen, and 15” each for left and right leg). The experimenter used one of the HTC-VIVE controllers to deliver the touches, while the participants looked at themselves (first-person perspective) while in their virtual bodies. Additionally, after the first-person perspective procedure (1PP), a mirror appeared on the wall in front of the avatar; the participants were asked to look at their avatars reflected in the mirror, while the same visuo-tactile procedure was repeated (mirror view). The complete duration of the visuo-tactile stimulation procedure was three minutes. 

The control group asynchronous condition followed the procedure just described (1PP and mirror view) but the experimenter delivered the continuous touches, with a two-second delay, so that the visual feedback was inconsistent between what participants saw and what they really felt. For example, when they were feeling the touch to their forearm, they saw their avatar being touched on the shoulders. 

**Figure 1:**
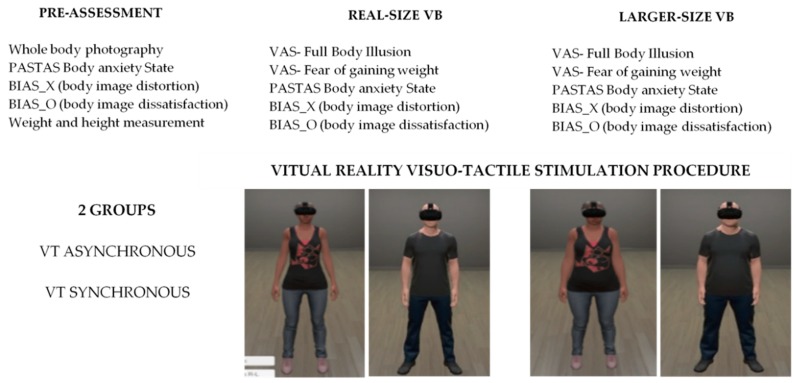
Experimental design scheme. Between-subjects group condition (synchronous versus asynchronous), within-subjects assessment condition (pre-assessment, real-size and larger-size virtual bodies), and dependent variables reported. VT = visuo-tactile, VB = virtual body, VAS = visual analog scales.

In sum, both groups (synch/asynch) initially answered the test battery in the pre-assessment, then all participants were exposed to two virtual bodies: the first with the same body size as the participant and the second one larger than the participant (see Figure 1). In each body size condition, a visuo-tactile stimulation procedure was applied, using first a 1PP and second using a mirror view. Then, once they had finished the visuo-tactile stimulation procedure, participants answered the VAS-FBI and VAS- FGQ questions orally. Finally, after the last of each body size exposures, they left the VR environment and answered the BIAS-BD and PASTAS questionnaires.

### 2.5. Statistical Analysis

The outcome of the intervention was analyzed with the statistical software IBM SPSS Statistics v.23. Mixed between (group)–within (assessment condition) analyses of variance (ANOVA) were conducted. The assumptions were partially met, since there was homogeneity of variances (*p* > 0.05) as assessed by the Levene’s test. The assumption of sphericity was not completely met (*p* < 0.05) for some variables (VAS_Fear of weight gain), therefore, it was decided to use the Green–House–Geisser corrected test for sphericity instead of this variable. Data were not normally distributed in all the variables assessed by the Kolmogorov–Smirnov test. However, it was decided to run the test regardless, as ANOVA is considered a robust test even in the case of a deviation from normality [35]. Finally, few outliers were detected in both BIDs measures, as assessed by inspection of a boxplot. Statistical analyses were conducted with and without the outliers, and since the results did not significantly differ, it was decided to include them in the analyses. 

## 3. Results

### 3.1. Descriptive Results

A two-way mixed ANOVA was conducted. Means and standard deviations of all dependent variables were specified for each experimental condition at the different assessment times (Table 1).

According to the descriptive results, in the pre-assessment, there were no statistically significant group differences (*p* > 0.05) in any of the body anxiety or BID measures. Therefore, it was decided to continue the statistical analyses.

### 3.2. Statistical Analyses

As shown in Table 2, there were no statistically significant interactions between group and assessment conditions (*p* > 0.05) on body dissatisfaction (BIAS_O), body distortion (BIAS_X), fear of gaining weight (VAS-FGW), and full body illusion (VAS-FBI). However, there was a significant interaction between group and assessment conditions in body anxiety (PASTAS) *F* (2, 96) = 3.297, *p* = 0.041, partial η^2^ = 0.064. 

**Figure 2:**
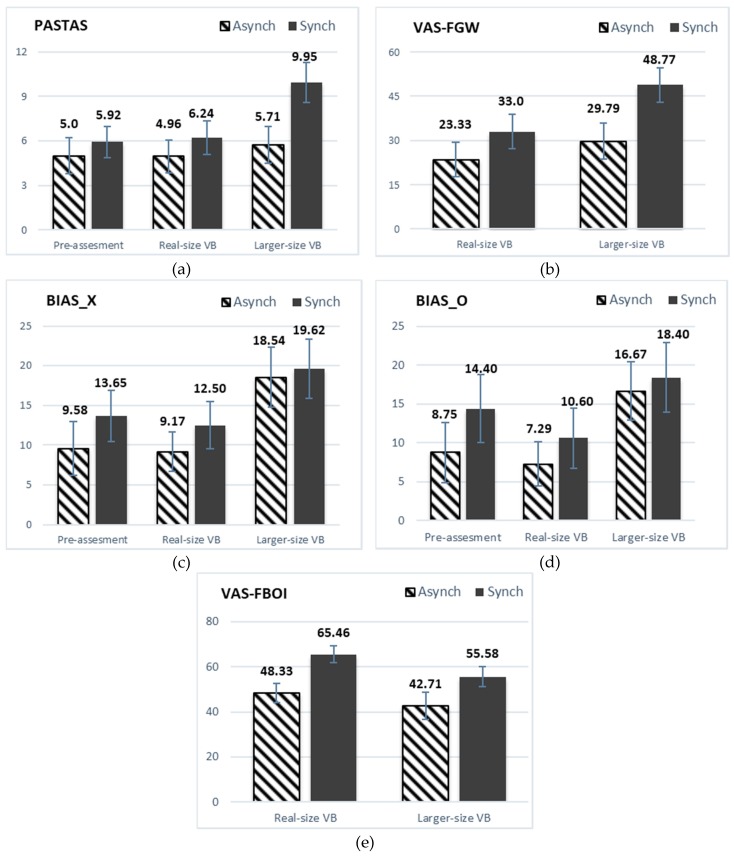
(**a**)Body anxiety mean scores, (**b**) fear of weight gain mean scores, (**c**) body image distortion mean scores, (**d**) body image dissatisfaction mean scores, (**e**) full-body ownership illusion mean scores. Means of the asynchronous and synchronous groups in the three assessment conditions (pre-assessment, real-size virtual body, larger-size virtual body). Error bars represent standard errors. Body image disturbances (Body Image Distortion-BIAS_X, Body Image Dissatisfaction-BIAS_O), Body anxiety (PASTAS), fear of gaining weight (VAS-FWG), and full-body ownership illusion (VAS-FBI). VB = Virtual body.

Regarding body anxiety (PASTAS), three additional between-subjects ANOVAs were conducted separately in each assessment condition to follow up the simple effects of group on assessment time. Mean differences (*MD*) ± standard error (*SE*) are specified. Body anxiety scores did not differ significantly between the two groups (*p* > 0.05) in the pre-assessment and the real-size virtual body conditions (see Figure 2a). With the larger size virtual body, even though differences between the groups did not reach statistically significance *F* (1, 48) = 3.492, *p* = 0.07, partial η^2^ = 0.068, with a medium effect size according to Cohen [36]; body anxiety scores were higher in the synchronous than in the asynchronous group condition (*MD* = 3.446 ± *SE* = 1.84). 

The main effect of assessment condition (see Table 2) showed that there were statistically significant differences (*p* < 0.05) in body anxiety (PASTAS), body image disturbances (BIAS_X or BIAS_O), fear of weight gain (VAS-FWG), and full body ownership illusion (VAS-FBI) measures.

To analyze the outcomes in more detail, multiple post-hoc tests (pairwise comparisons) were conducted. Mean differences (*MD*) ± standard error (*SE*) were specified. As can be seen in Table 3, all the measures were significantly higher in the larger-size VB condition than in both the real-size VB and the pre-assessment (for illustrations see Figure 2a–d). However, the VAS-FBI scores decreased compared with the real-size VB. 

Finally, there was a statistically significant main effect of group on the VAS-FBI F (1, 48) = 7940, p = 0.007, partial η^2^ = 0.142. Regardless of the assessment condition, the synchronous group showed higher levels of FBI than the asynchronous group (see Figure 2e). 

## 4. Discussion

This study aimed to assess whether a visuo-tactile embodiment-based procedure could elicit changes in the body anxiety and body image disturbance responses of healthy participants when owning two different-size virtual bodies. In addition, two different visuo-tactile group conditions were compared (synchronous versus asynchronous visuo-tactile stimulation).

As expected, all participants, regardless of group, showed higher levels of body image disturbances (BIDs) and body anxiety after owning the larger-size virtual body (VB) than after owning with the real-size VB, and the scores were higher in the synchronous visuo-tactile group. Our results support previous findings suggesting that body image is a state rather than a trait, and can, thus, be modified by internal and external stimuli [6], such as owning a larger-size VB. Jakatdar, Cash, and Engle [37] suggested that malleability of the body image is more frequent in ED patients but also occurs in healthy participants, as our data show. Our results contrast with those of previous studies which did not report significant differences on body size estimating tasks before and after owning a larger mannequin/virtual body [15,17]. The differences in the results may be explained by differences in the methodological procedures used. For instance, even though in all the studies there was a larger-size/overweight virtual body condition, in our study, participants previously owned a real-size virtual body that represented their own silhouette, while in two previous studies [15,17], thin/underweight virtual bodies or mannequins were used instead. 

In addition, the appearance of the virtual body, i.e., the body shape, may also explain the differences between the present study and previous studies. The appearance of the virtual body may play an important role not only in the eliciting of the illusory feelings of ownership [38,39], but also in eliciting emotional responses in healthy participants [15]. In our study a weight gain proportionally spread over the whole body (e.g., the belly, thighs, waist, arms) was reproduced, resulting in a more realistic increase of weight across the body, and consequently evoking higher anxiety and body image disturbance levels in participants when owning the larger virtual body than when owning the real-size virtual body. In contrast, other studies may have not found similar results because the weight gain was represented less proportionally, for example, by increasing only the stomach size of the mannequin [15]. Additionally, the increase in the weight of our avatar was previously based on the real-size virtual body with the participant’s silhouette, allowing a more accurate and natural weight increase in the larger-size virtual body. 

Regarding body anxiety levels, our results are in line with those of a previous study by our group [31], in which healthy college students undergoing a synchronous visuomotor procedure reported higher levels of body anxiety and fear of weight gain after owning a larger-size VB than after owning their real-size VB. However, the current study has attempted to overcome some of the limitations reported there, such as the small sample size, the lack of an asynchronous visuomotor procedure, and the lack of a baseline pre-assessment time condition. Furthermore, BID and body anxiety levels did not differ significantly after owning a virtual avatar with participants’ real measurements (real-size VB) compared with the pre-assessment. This suggests that, regardless of the type of visuo-tactile stimulation, all participants delivered, perceived, and felt the real-size VB as their own body, even without being aware of it. 

According to the differences between the synchronous and asynchronous visuo-tactile groups, there were not statistically significant group differences in BID and body anxiety measures. Interesting non-significant tendencies were found in body anxiety measures. Participants who underwent a synchronous visuo-tactile procedure reported higher body anxiety and fear of gaining weight levels than those in the asynchronous condition, at the larger size VB. One possible explanation for these tendencies, is that some of the body parts that were (in)congruently stimulated (e.g., the stomach or the thighs) are usually reported as body areas of higher concern [40] or weight-related body areas [33]. Previous studies have reported that individuals with ED [40,41] show an attentional preference towards these self-reported unattractive body areas. Therefore, it might be expected that a synchronous visuo-tactile stimulation over those salient body areas with a significant weight gain might elicit a higher more unpleasant anxious response that was not observed at equal levels in the asynchronous group. Consequently, our results suggest that the specific influence of the FBI induced by a synchronous visuo-tactile stimulation over specific body parts may differentially affect body anxiety and BID measures after owning a larger-size VB. Regarding BID measures, our results are in line with previous studies in which inducing a synchronous or asynchronous visuo-tactile stimulation did not affect body distortion levels after owning a skinny virtual body [18], or an underweight or overweight virtual body [17]. 

Regarding the FBI levels, the synchronous visuo-tactile group showed significantly greater FBI levels in both the real-size VB and larger-size VB than the asynchronous group. Our results are in line with previous studies that compared synchronous versus asynchronous visuo-tactile stimulation procedures for eliciting FBI by owning either a slim virtual body or mannequin body (e.g., [15,16,21,23]) or an overweight virtual body [15,17,22]. However, some important differences between our study and previous research should be noted, such as, for example, the type of visual perspective displayed. In the present study, a combination of both 1PP and a mirror view perspective were used successively in order to strengthen the FBI, in comparison to previous studies that have used only a first-person perspective to elicit the FBI (e.g., [15,21,23]). 

Another important difference is the sort of tactile stimulation delivered. A visuo-tactile stimulation procedure over the whole body (abdomen, upper and lower extremities) was delivered in this study, in contrast to previous studies in which the only stimulated body parts were the abdomen or the belly [18,23], or the upper and lower limbs [17]. Additionally, the body posture was also different from the study conducted by Piryankova et al. [17], in which the participants were seated while the upper and lower virtual limbs were touched. In our study, all participants were in a standing position, in which the realism of the virtual avatar is less likely to be affected.

The current study presents some limitations that should be addressed in future research. Body mass index (BMI) was not controlled. Although we excluded participants with obesity (BMI > 30) or who were moderate-to-severely underweight (BMI < 17), previous literature suggested that overweight, compared to healthy weight, individuals present higher body dissatisfaction levels [42]. Another important limitation is that no screening questionnaire or clinical structured interview was used to properly assess the presence of an ED diagnosis or other mental disorders. It should be noted that even though our participants were allocated randomly, the synchronous visuo-tactile group reported slightly higher BID levels than the asynchronous group at the pre-assessment time condition. However, as the group differences did not reach significance, it was decided to continue the statistical analyses. As regards the length of the experiment, participants generally spent one hour in the laboratory. We realize that it may cause fatigue, especially because participants were asked to stand still while the experimenter was running the tactile-stimulation procedure for each body size condition. Following the previous argument, and so as not to lengthen the experiment further, it was decided not to include other body-size conditions such as a slimmer virtual body, as the studies by Preston et al. [15] or Piryankova et al. [17] had done. 

These results may have different implications in clinical practice. In the near future, it would be interesting to apply FBI and VR embodiment-based techniques to treat the fear of gaining weight and body anxiety in ED patients, and specifically in anorexia nervosa (AN) patients. One possibility would be to develop an exposure therapy to one’s own body, in which an AN patient could own an avatar with their own body silhouette and their BMI would increase gradually, according to a pre-established hierarchy. Additionally, our results support those of previous studies suggesting that VR embodiment-based techniques may be a useful tool for interventions that aim to modify or improve BID levels among healthy participants, or in clinical ED patients (e.g., [23]).

Another interesting approach would be to use VR embodiment-based procedures not only to treat BID, but also to assess it. Following a similar procedure to paper-based figural drawing scale questionnaires (e.g., BIAS-BD, [12]) or human-based 3D computer scales [43,44], participants or ED patients could own a virtual avatar and modify different body parts (or the whole body of the avatar) according to their perceived or desired body size. Additionally, using a VR assessment procedure would also allow the generation of more ecologically valid experimental settings that represent daily life situations: for instance, looking at one’s own body in a mirror. 

## 5. Conclusions

This study provides new evidence of the usefulness of VR embodiment-based techniques to induce changes in body image disturbances or body anxiety in a non-clinical sample of college students. Specifically, our findings provide new information about how the influence of the FBI, induced by a synchronous visuo-tactile stimulation, may not only strengthen the FBI when participants own their real-size VB, but may also elicit higher BID and body anxiety levels after owning a larger-size VB, with body anxiety measures being particularly affected by a congruent visuo-tactile stimulation. In addition, a new VR visuo-tactile stimulation procedure over the whole body, which combines first-person perspective and mirror view, is presented and discussed. Finally, this study presents a novel procedure for adapting our virtual avatar bodies based on the real-size frontal silhouettes of our participants, allowing the recreation of a realistic real-size virtual avatar, but even more importantly, the simulation of a more realistic weight gain in our virtual bodies. 

The use of VR embodiment-based techniques opens up new possibilities in body image research, some of which are presented and briefly discussed in this article. However, considering the high prevalence of BID among ED patients [12] and among non-clinical populations [10,11], future interventions should continue to focus on understanding and improving the disturbed representations that so many individuals have of their body appearance. 

## Figures and Tables

**Table 1:** Descriptive outcomes, means, and standard deviations stated in the visuo-tactile group condition (asynchronous group versus synchronous group) at the different assessment conditions.

	Pre-Assessment		Real-Size VB		Larger-Size VB	
	Asyn-Group *n* = 24 M (SD)	Syn-Group *n* = 26 M (SD)	Asyn-Group *n* = 24 M (SD)	Syn-Group *n* = 26 M (SD)	Asyn- Group *n* = 24 M (SD)	Syn-Group *n* = 26 M (SD)
PASTAS	5.00 (5.87)	5.92 (5.31)	4.96 (5.46)	6.23 (5.77)	5.71 (6.07)	9.15 (6.89)
BIAS_X	9.58 (16,68)	13.65 (16.40)	9.17 (12.22)	12.50 (15.25)	18.54 (18.56)	19.62 (18.92)
BIAS_O	8.75 (18.72)	14.40 (21.81)	7.29 (14.06)	10.60 (19.22)	16.67 (18.57)	18.40 (22.39)
VAS-FBI	-		48.33 (20.44)	65.46 (19.15)	42.71 (28.97)	55.58 (23.07)
VAS-FGW	-		23.33 (26.77)	33.00 (29.34)	29.79 (29.39)	48.77 (29.48)

Note: Body Image Disturbances (Body Image Distortion-BIAS_X, Body Image Dissatisfaction- BIAS_O), Body Anxiety (PASTAS), fear of gaining weight (VAS-FWG), and full-body illusion (VAS-FBI). VB = Virtual body, Async = Asynchronous, Sync = Synchronous group conditions.

**Table 2:** Mixed between–within subject analysis of variance comparing groups (Group 1-Asynchronous group versus Group 2-Synchronous group) with the different assessment conditions.

	Time x Group			Time of Assessment		
	*F*	*p*	η^2^	*F*	*p*	η^2^
PASTAS	3.297	0.041 *	0.064	8.554	0.001 *	0.151
BIAS_X	0.347	0.707	0.007	11.798	0.001 *	0.197
BIAS_O	0.410	0.665	0.009	8.177	0.001 *	0.148
VAS-FBI	0.312	0.579	0.006	4.130	0.048 *	0.079
VAS-FGW	3.855	0.055	0.074	21.968	0.001 *	0.314

Note: Green–House–Geisser corrected test for sphericity used. * Significant *p*-values < 0.05.

**Table 3:** Post-hoc analyses (pairwise comparison) at the different assessment times (pre-assessment, real-size virtual body and larger-size virtual body).

	Assessment Time Conditions					
	Pre-Assessment versus Real-Size VB		Larger-Size VB versus Real-Size VB		Larger-Size VB versus Pre-Assessment	
	MD	SE	MD	SE	MD	SE
PASTAS	0.133	0.491	1.837 *	0.517	1.970 *	0.584
BIAS_X	0.785	1.717	8.245 *	1.957	7.460 *	1.938
BIAS_O	2.629	1.915	8.588 *	2.133	5.958	2.448
VAS-FBI			−7.755 *	3.816		
VAS-FGW			11.114 *	2.371		

Note: MD = Mean differences, SE = Standard Error. * Significant *p*-values < 0.05. VB = Virtual body.
